# Wearable Sweat Biosensors Refresh Personalized Health/Medical Diagnostics

**DOI:** 10.34133/2021/9757126

**Published:** 2021-10-22

**Authors:** Wenhui Ji, Jingyu Zhu, Wanxia Wu, Nanxiang Wang, Jiqing Wang, Jiansheng Wu, Qiong Wu, Xuewen Wang, Changmin Yu, Gaofeng Wei, Lin Li, Fengwei Huo

**Affiliations:** ^1^Key Laboratory of Flexible Electronics & Institute of Advanced Materials, Nanjing Tech University, Nanjing 211816, China; ^2^Frontiers Science Center for Flexible Electronics, Xi'an Institute of Flexible Electronics (IFE) and Xi'an Institute of Biomedical Materials & Engineering, Northwestern Polytechnical University, Xi'an 710072, China; ^3^Naval Medical Department, Naval Medical University, Shanghai 200433, China; ^4^The Institute of Flexible Electronics (IFE, Future Technologies), Xiamen University, Xiamen 361005, China

## Abstract

Sweat contains a broad range of critical biomarkers including ions, small molecules, and macromolecules that may indirectly or directly reflect the health status of the human body and thereby help track disease progression. Wearable sweat biosensors enable the collection and analysis of sweat *in situ*, achieving real-time, continuous, and noninvasive monitoring of human biochemical parameters at the molecular level. This review summarizes the physiological/pathological information of sweat and wearable sweat biosensors. First, the production of sweat pertaining to various electrolytes, metabolites, and proteins is described. Then, the compositions of the wearable sweat biosensors are summarized, and the design of each subsystem is introduced in detail. The latest applications of wearable sweat biosensors for outdoor, hospital, and family monitoring are highlighted. Finally, the review provides a summary and an outlook on the future developments and challenges of wearable sweat biosensors with the aim of advancing the field of wearable sweat monitoring technology.

## 1. Introduction

Medical health is important to every individual and is associated with social and economic development. Currently, countries all over the world have established a passive modern medical health treatment system, exemplified by hospitals, through which people can obtain information about their health condition [1]. Generally, people visit hospitals to receive professional diagnosis from doctors only after they notice obvious symptoms, rendering it extremely difficult to prevent disease occurrence in advance. In addition, the health information acquired during reactionary treatment is discontinuous and imprecise, thus failing to accurately prevent and track diseases. Several traditional health devices that can offer preventive applications, such as stethoscopes and blood pressure monitors, require specialized personnel to operate and cannot be used for long-term dynamic tracking or diagnosis of chronic diseases.

Wearable biosensors are designed to achieve continuous, real-time, noninvasive monitoring of physiological indicators of the human body by attaching biosensor elements to a flexible substrate and placing them closely against the skin or clothes, enabling individuals to obtain dynamic and personalized health information at any time [2, 3]. Wearable biosensors are typically divided into two categories: (A) sensors based on physiological signal monitoring, such as tracking of blood pressure [4], cardiac activity [5], and temperature [6], and (B) wearable sensors based on biochemical parameter monitoring, such as dynamic tracking of metabolites in biological fluids, including sweat [7], tears [8], and interstitial fluids (ISF) [9]. Compared with wearable sensors for physiological signal monitoring, wearable sensors based on biochemical parameter monitoring provide a larger-scale overview of an individual's health status and more dynamic molecular-level information [10, 11]. Among measurable body fluids, only sweat can be monitored noninvasively, continuously, and in real time. Additionally, it is possible to precisely control the location and amount of sweat produced by local chemical stimuli of the body surface according to the application [12]. Sweat contains a wealth of biochemical information, and variations in metabolite concentrations of sweat allow for the assessment of dehydration, substance abuse, stress, and disease progression [13].

**Scheme 1:**
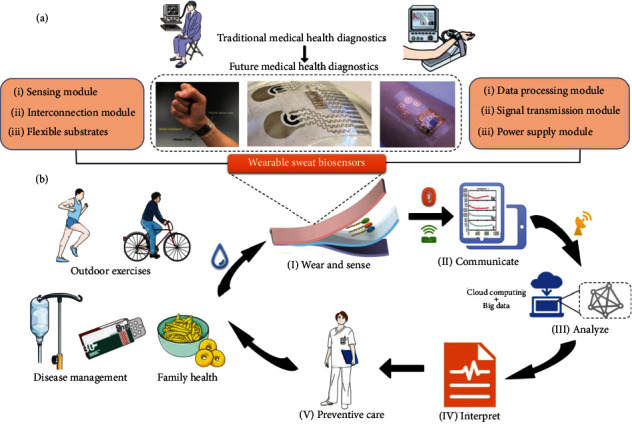
Schematic diagram of the components of a wearable sweat biosensor and personalized medical diagnosis process. (a) From traditional medical health diagnostics to future-oriented medical health diagnostics based on wearable sweat biosensors and compositions of wearable sweat biosensors, including sensing module, interconnection module, flexible substrates, data processing module, signal transmission module, and power module. (b) Wearable sweat biosensors for outdoor sports, hospital, and family health management applications. The sensor worn by a person can monitor the changes of biochemical substances in sweat and transmit the test signal to a smartphone *via* Bluetooth or near-field communication (NFC), and the biochemical signal can be transformed into a readable electrical/optical signal using software on the phone. Combined with big data and cloud computing to analyze the data and predict future health levels, then the generated reports are transmitted to a physician to provide targeted medical care.

The wearable sweat biosensor, which incorporates flexible epidermal electronics, microfluidic chip technology, electrochemical technology, and other multidisciplinary frontiers, enables the continuous collection, detection, and transmission process of human epidermal sweat and has initially yielded a number of innovative advances in the field of human physiological information monitoring, revealing the potential future value of disruptive applications in the medical health field [14–16]. Applicable to different scenarios such as sports and fitness, daily work, occupational disease monitoring, postoperative tracking, chronic disease management, and elderly care with sweat biomarker detection at the core, wearable sweat biosensors can be developed to monitor the physiological health indicators. Realizing real-time noninvasive monitoring of an individual's daily health status or disease development will not only enable accurate monitoring and management of various chronic diseases but also provide advanced preventive health care, ultimately reducing the probability of developing a disease and improving health (Scheme 1).

In recent years, with the rapid advancement in technologies of flexible electronics, chemical sensing, and hardware integration, the shortcomings of some wearable sweat biosensors have been overcome [17]. This review will focus on the application of wearable sweat biosensors in different scenarios, including continuous health and disease monitoring, together with information on the physiological/pathological parameters associated with sweat.  Section 1 introduces the background of wearable sweat biosensors.  Section 2 summarizes sweat production, metabolite secretion mechanisms, and the composition of sweat.  Section 3 introduces the composition of the wearable sweat biosensors and common preparation methods.  Section 4 presents the latest applications of wearable sweat biosensors in three scenarios: outdoor, hospital, and family monitoring.  Section 5 outlines the future development and challenges of wearable sweat biosensors.

## 2. Metabolites in Sweat and Related Diseases

### 2.1. Sweat Production

Sweat is a fluid secreted by the sweat glands of humans and is transported to the epidermal surface through the secretory ducts. Sweat glands are distributed in the dermis of the skin throughout the body and are divided into large sudoriferous glands and eccrine glands [18]. Large sudoriferous glands are mainly located in the axillae, while eccrine glands are distributed throughout the epidermis. Normally, sweat is slightly acidic, with a pH of 4.0-6.8 [19, 20]. According to previous studies, during exercise or thermal stimulation, the body increases heat dissipation by evaporation of sweat from the body surface. Sweat is also produced during stress or excitement.

There are two categories to stimulate sweat production: passive and active. Passive stimulation often occurs due to hot or humid weather and is used to maintain normal body temperature by evaporating sweat to decrease the body heat. In contrast, the sweat that comes from the active movement is called active sweating. It not only sustains the normal body temperature but also carries away a small amount of waste products generated within the body during exercise. If sweat is needed for specific analysis, the user must exercise vigorously or otherwise raise body temperature in order to produce sweat from most parts of the body, which is excessively time-consuming [21]. Therefore, external stimulation is an effective and rapid alternative to facilitate sweat production. External stimulation methods include iontophoresis, reverse iontophoresis, and drug stimulation. Iontophoresis is a noninvasive method, in which a low current drives a drug with the same quantity of electrical charges to the surface of the skin and triggers the secretion of sweat from nearby glands [22]. Reverse iontophoresis uses electroosmosis to drive tissue fluids from the epidermis to the skin surface. Specifically, a low current induces the flow of positively charged ions from the anode to the cathode, which may cause skin discomfort [23]. The drug stimulation method involves the application of several chemical drugs to induce the production of sweat. For example, iontophoretic therapy with carbachol stimulates the local secretion of sweat, which lasts much longer than that stimulated by trichothecene [24].

### 2.2. Secretion and Composition of Sweat

Sweat contains abundant biochemical information that may reflect the status of the organism at the molecular level. To understand the relationship between the changes in sweat analyte concentration and physiological function, it is necessary to be aware of the mechanism of analyte secretion. Sweat is primarily derived from the exocrine glands, which consist of secretory coils responsible for producing sweat and of ducts that transport sweat to the skin surface [25]. During this process, analytes such as electrolytes, metabolites, proteins, and peptides are distributed in the sweat. Sodium ions (Na^+^) and chloride ions (Cl^−^) are actively transported between the blood and the secretory coils, creating an osmotic pressure difference that allows sweat to pass into the sweat glands [26].

A number of chemicals found in sweat, including metabolites (e.g., glucose [27], urea [28], lactate [29], cortisol [30], and ethanol [31]), electrolytes (e.g., potassium (K^+^) [32], Na^+^ [33], Cl^−^ [29, 34], ammonium (NH_4_^+^) [31], zinc (Zn^2+^) [35], and copper (Cu^2+^) [36]), and macromolecules (e.g., peptides [37], proteins [38], and cytokines [39]), are closely related to multiple diseases, allowing pathogenesis to be tracked by monitoring variations in the concentration of these substances. For example, the concentration range of Na^+^ under normal physiological conditions is 0.23-2.29 mg/mL, but a large loss of Na^+^ in patient sweat may indicate hyponatremia [33]. The natural concentration of K^+^ is 0.04-0.72 mg/mL, which will fluctuate during electrolyte disturbances [32]. Levels over 0.45-1.8 mg/mL of sweat lactate are generally regarded as a biological criterion for inadequate oxidative metabolism and local ischemia [29]. Additionally, sweat glucose concentration is associated with blood concentration and serve as a clinical diagnosis of diabetes, while Cl^−^ level can be regarded as a diagnostic criterion for cystic fibrosis (CF) in the newborns [27, 40]. The detection of biochemical substances in sweat presented in this review, especially in Section 4, is listed in Table 1.

At present, the specific secretion mechanisms of several analytes are still not fully investigated; therefore, further research and development of secretion mechanisms or models for sweat analytes are needed to lay the foundation for wearable sweat biosensors.

## 3. Wearable Sweat Biosensors

Continuous, dynamic, and noninvasive monitoring of the biochemical components of sweat requires wearable sweat biosensors to interact directly with the skin surface for continuous collection and accurate analysis of sweat. The resulting information will be readily converted into readable electrical/optical signals and transmitted to the user or physician *via* a wireless transmission device, ultimately providing the dynamic health information of the wearer.

**Figure 1:**
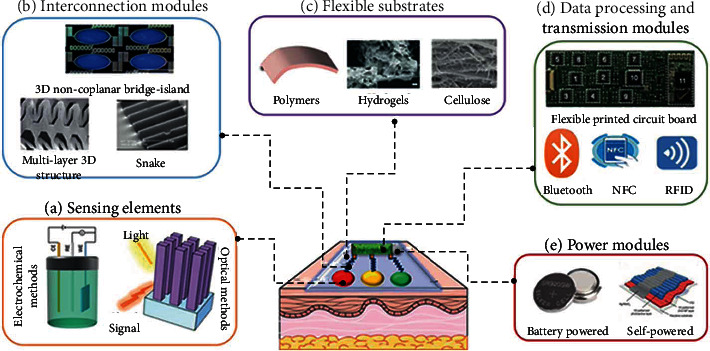
Illustration of the five compositions of wearable sweat biosensors: sensing elements, interconnection modules, flexible substrates, data processing and transmission modules, and power modules.

In this section, we summarize a wearable sweat biosensor with sensing elements, interconnection modules, flexible substrates, data processing and transmission modules, and power supply modules (Figure 1). In addition, common preparation methods are also described.

1. Sensing elements. The accurate detection of sweat on the skin surface demands rapid response, high sensitivity, great selectivity, and stability of the sensing module. Currently, the detection principles of the sensing module can be broadly classified into electrochemical and optical sensing [41]

Electrochemical methods are used to generate electrical signals by the specific redox reaction of the analytes on the electrode, thus enabling the conversion of biochemical information into readable electrical signals, offering high sensitivity, high selectivity, and fast response time [42]. In recent years, different electrochemical methods have been implemented for wearable sweat biosensors, including potentiometric, amperometric, voltammetric, and impedance methods [43]. Here, we introduce two commonly applied electrochemical detection methods: potentiometric and amperometric methods. Potentiometric methods are often applied to detect ions and usually consist of an ion-selective electrode (ISE) as the working electrode (WE) and Ag/AgCl as the reference electrode (RE). The variation between the voltage of the ISE and the concentration of the ion to be measured is in accordance with the Nernst equation [44]. Amperometry is typically performed to determine the current generated when an analyte undergoes a redox reaction at a bias voltage and is usually composed of a three-electrode system with a WE, a RE, and a counter electrode (CE) [45]. Electrochemical methods for the detection of different metabolites in sweat use different detection methods and principles, and we will briefly describe the principles of measurement of ions, small molecules, and macromolecules. For the detection of ions, the prepared ion-selective membrane is usually modified on the WE by spin coating or drop coating methods, and the change in ion concentration is detected by the open-circuit potential test (OCPT) [32, 33]. The ion-selective membrane contains specific carriers for the recognition of ions; e.g., valinomycin is commonly used for K^+^. The detection of small molecules is usually achieved by modifying nanomaterials with excellent catalytic and conductive properties on the surface of the WE [46]. Small molecules (e.g., dopamine, urea, and ascorbic acid) are able to undergo redox reactions at lower bias voltages, and the current generated is recorded by the chronoamperometry (CA), differential pulse voltammetry (DPV), square wave voltammetry (SWV), and cyclic voltammetry (CV), thus enabling the detection of small metabolites. Detection of large molecules is usually done by modifying biological materials with specific recognition such as enzymes, antibodies, and aptamers on the surface of the WE, which is recorded by electrochemical impedance spectroscopy (EIS) [30].

Optical sensors based on colorimetric or fluorescence methods have drawn more interest of researchers for their simpler preparation process, lighter and thinner appearance, and no involvement of the power supply units, which allow them to be more easily incorporated into wearable sensors than electrical alternatives [47–50]. The detection mechanism of optical sensors is a chemical reaction between the analyte and a chromogenic substance that produces a quantifiable change under the excitation or irradiation of light, which enables quantitative or semiquantitative analysis by the naked eye or visual readout devices. For example, simultaneous detection of Cl^−^ and pH in sweat was achieved using silver chloranilate and pH-sensitive dyes [51]. In addition to these conventional dyes, several emerging fluorescent probes, quantum dots, and metal nanoparticles can also be applied to wearable sweat optical biosensors in the future. These methods offer simple preparation, cost-effectiveness, portability, and light weight, but their accuracy is slightly lower than that of electrochemical methods. Because the color interpretation may be affected by the eye or the color readout device, this can potentially lead to biased results. Colorimetric or fluorescence methods may bring advantages to environments where accuracy is not essential.

(2) Interconnection modules. The interconnection module of wearable sweat biosensors undertakes a crucial role between the sensing module and the signal processing module, owing to the fact that it is the essential component to mitigate mixing parameters and ensure high signal-to-noise ratio and effective signal transmission [52]. Researchers have tried various structures and preparation procedures to reduce the susceptibility of the interconnection modules to external factors such as stretching and bending [53]. There are also a variety of existing interconnection module designs, such as snake structures [54, 55], fish scale structures [28], multilayer 3D structures [56], and 3D noncoplanar bridge-island structures [57]. Therefore, the design of the interconnection modules has progressed from a pair of lines and spiral and serpentine structures to complex flower-like and basket-like structures, which have led to a breakthrough in the stretchability and flexibility of the interconnection modules [58]
(3) Flexible substrates. A flexible substrate is a key element to wearable sensors, as it assumes a close, comfortable, and soft adhesion to the skin surface. A flexible substrate is usually required to possess good stretchability, good mechanical properties, breathability, and biocompatibility. To satisfy the requirements of flexible electronic devices, flexible substrate materials should be thin, flexible, and stretchable and have favorable insulation and corrosion resistance, biocompatibility, and self-healing ability. Frequently available flexible substrates can mainly be classified as polymers, hydrogels, and cellulose. Various polymer films, including poly(styrene-ethylene-butadiene-styrene) rubber (SEBS) [59], polyethylene terephthalate (PET) [60], and polydimethylsiloxane (PDMS) [61], have been widely used as substrates for electronic devices and flexible electrodes because of their excellent mechanical properties, chemical resistance, and thermal stability. Flexible substrates of hydrogels and aerogels are also widely employed in wearable biosensors due to their high flexibility, stretchability, and tunability [62]. In addition to polymer and hydrogel materials, cellulose-based substrates, such as paper-based or textile materials, are now being widely used in the preparation of wearable devices [63]. Paper-based substrates are of interest to scientists because of their cost-effectiveness, available applications, biocompatibility, and capillary effect [64]. Textiles represent a special class of flexible and stretchable material substrates, including cotton fabrics [65] and silk with excellent flexibility and deformability [66–68]. Due to the softness and curvature of the human surface, wearable sensors need to remain in seamless contact with the human surface during daily activity or movement; therefore, excellent stretchability is a vital parameter for flexible substrates [69]. Two strategies are commonly used to achieve stretchability in wearable sensors. The first approach is to bond thin conductive materials with low Young's modulus directly to a flexible substrate. The second approach is to use intrinsically stretchable conductors, specifically by mixing conductive substances into a flexible substrate to provide great stretchability and conductivity. For example, Wang et al. modified a quaternary hydroxyethyl cellulose hydrogel layer (Young's modulus: ~14.2 kPa) on a sodium lignosulfonate-borax hydrogel substrate (Young's modulus: ~101.3 kPa) by a coating method, resulting in a hydrogel with stable sensing properties and excellent tensile properties [70]. Among commonly used polymeric substrates, polytetrafluoroethylene resin as a plasticizing additive can enable silicone substrates to withstand strains of up to 188% [71], while cross-linking polyvinyl alcohol with sodium borate has been reported to produce a highly stretchable, transparent conductive gel that can withstand strains of up to 700% [72], even though the stretching properties of the flexible substrate can be adjusted by filling it with some special materials, which may also affect other properties. Therefore, the design and synthesis of flexible materials with excellent stretchability, ductility, electrical conductivity, and biocompatibility will remain a critical concern in the future
(4) Data processing and transmission modules. Data processing and signal transmission involve extracting, transforming, analyzing, and integrating optical or electrical signals from biosensors and transmitting data from one electronic device to the other to be readable for the user [73]. A commonly used data processing element is the flexible printed circuit board (FPCB), which allows efficient processing of data while maintaining the independence and selectivity of individual sensor operations [6]. The common transmission modules currently used in wearable devices are Bluetooth, near-field communication (NFC), and radio frequency identification (RFID). Bluetooth can communicate over a maximum distance of approximately 100 m and transmit more data than the other options and has superior advantages of being radiation-free, environmentally friendly, and user-friendly [74]. Another commonly used transmission technology is NFC, which operates at 13.56 MHz and has a communication distance of up to 20 cm [75]. Its maximum data transfer rate is much lower than the Bluetooth, but devices do not need to be paired in advance, and the connection between two NFC can be established within only 0.1 s. There is also a type of RFID technology, which relies on electromagnetic waves to transmit signals; typical RFID transmission time is extremely short, typically less than 100 milliseconds [72]. RFID with high-frequency capability can simultaneously verify and access multiple information profiles, which greatly facilitates transmission performance
(5) Power modules. Wearable sweat biosensors based on electrochemical methods are the most commonly available models, thereby rendering the power supply an essential component. Currently, most developed wearable sensors rely largely on commercial batteries for uninterrupted power supply, but their bulk and surface areas are large [2]. Therefore, self-powered, rechargeable power supplies or power supply modules that attenuate the need for external power supply are becoming increasingly popular. Theoretically, the energy obtained from body heat, exercise, and sunlight is sufficient to power the wearable biosensors. Unfortunately, wearable sensors require a stable power source to work, yet most power modules used for self-powering are vulnerable to external factors such as changes in sunlight intensity, human motion intensity, and body heat emissions [76]
(6) Common preparation methods. The popular preparation approaches for wearable sensors include photolithography and printing methods. Photolithography refers to the transfer of the pattern on the mask plate to the substrate with the help of photoresist under the action of light [39]. Common printing methods are inkjet printing [46], screen printing [30], and 3D printing [77]. Although photolithography is now relatively mature, its complex preparation process, high cost, and wasteful raw materials limit its application in large-area metal patterning. Therefore, with the evolution of preparation technology, several new technologies have been emerging. Inkjet printing is a contactless, pressure-free printing technology that does not require photolithography plates. Its printing accuracy can reach 20-30 microns, which can achieve high precision and high resolution, and it also has the advantages of fast speed, low cost, batch manufacturing, and flexible pattern shape design. With the rapid development of the electronics industry in recent years, environmental protection and cost considerations have promoted the development of screen printing. Screen printing is a mass printing method achieved by pressing ink through a patterned screen printing plate with a rubber squeegee. The resolution of screen printing is highly dependent on the fineness of the screen printing plate, but the substrate and the printing conductive ink are also important influencing factors. As a traditional flat printing technology, screen printing is widely used in the field of flexible electronics due to its simplicity, ease of patterned printing, and low cost. 3D printing is an emerging technology, also known as additive manufacturing. It builds objects by printing layer by layer using various bondable materials based on digital models. Commonly used printing materials are generally classified into metallic materials (e.g., silver nanowires) and nonmetallic materials (photosensitive resins). Metallic materials are mainly applied to 3D printing with sintering and melting as the manufacturing principle such as selective laser sintering (SLS) and selective laser melting (SLM). Nonmetallic materials are widely adopted in printing methods such as direct ink writing (DIW) and fused deposition molding (FDM). The sensor prepared by 3D printing has more accurate microstructure and better performance, filling the gap between traditional technologies such as coating and screen printing in the field of processing complex 3D structures, but it also has some disadvantages, such as the inability to achieve fine processing, limited types of printing materials, and high energy consumption

## 4. Applications in Different Scenarios

Wearable sweat biosensors with multifunctional and novel structural designs are promising platforms for monitoring a wide range of biochemicals in human sweat. The biochemicals in sweat fluctuate under different environmental conditions, and accurate, real-time, dynamic, and noninvasive monitoring of biochemical variations will contribute to a more comprehensive insight into human health information. Considerable efforts have been dedicated to the optimization of device design and the enhancement of the sensing performance of wearable sweat biosensors. In this section, we introduce the latest wearable sweat biosensor applications for outdoor, hospital, and family monitoring.

### 4.1. Outdoor Monitoring

**Figure 2:**
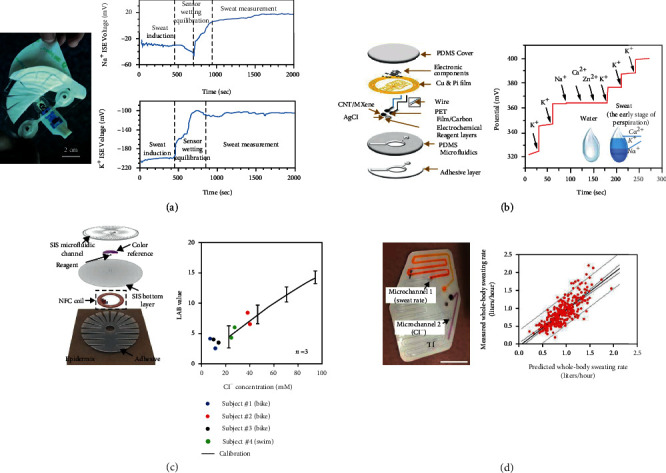
Outdoor monitoring. (a) Image of assembled wearable sweat patch and measurement results of Na^+^ and K^+^ in sweat produced by subjects during cycling. Reproduced with permission from Ref. [16], copyright 2018, The Royal Society of Chemistry. (b) Schematic diagram of the structure of the microfluidic integrated electrochemical sensing sweat patch and potential response for continuous and selective experiments of K^+^. Reproduced with permission from Ref. [79], copyright 2020, Elsevier B.V. (c) Schematic illustration of the waterproof, skin-like microfluidic/electronic device and the comparison of sensor and laboratory colorimetric detection. Reproduced with permission from Ref. [80], copyright 2019, American Association for the Advancement of Science. (d) Schematic diagram of the microfluidic patch and scatter plot of predicted versus actual whole-body sweat rate data under test conditions with different factors. Reproduced with permission from Ref. [81], copyright 2020, American Association for the Advancement of Science.

Body sweating due to strenuous exercise after intensive training and sports fitness may trigger electrolyte disturbances that can lead to physical damage. Athletes and physicians also desire the ability to monitor electrolytes, hydration, and muscle fatigue during exercise in order to develop training and rehabilitation programs. Hence, they need a method to continuously monitor sweat loss and composition in real time in an exercise environment [78]. Alizadeh et al. showed a fully integrated wearable perspiration biosensor device that allowed real-time monitoring of electrolyte concentration changes in the human body during strenuous exercise (Figure 2(a)) [16]. The conductive carbon and insulating medium layers were printed on the polyester substrate through the printer screen to prepare the electrodes, and then, poly 3,4-ethylenedioxythiophene (PEDOT) was electrodeposited to form a solid contact layer. The dynamic response of the sensor was tested for 100 mM salt solutions at flow rates of 1-20 *μ*L/min and for various salt concentrations at flow rates of 5-10 *μ*L/min, enabling clear detection of Na^+^. The biosensor was also successfully implemented to monitor real-time changes in Na^+^ and K^+^ during high-intensity cycling and moderate-intensity treadmill training.

Parker et al. prepared a highly sensitive, battery-free, and wirelessly transmitted electrochemical sensing patch for monitoring K^+^ in human sweat [79]. The WE of the biosensor was prepared by layer-by-layer deposition of multiwalled carbon nanotubes and MXene-Ti_3_C_2_TX hybrid solution on a PET substrate by screen printing. By adding carbon nanotubes, an intercalation and bridging effect is formed in MXene-Ti_3_C_2_TX nanosheets, which increases the carrier transport path and conductivity. The hybrid multidimensional network enables the sensor to obtain high surface activation area and faster charge transfer rate, which can strongly adsorb the valinomycin membrane to increase the K^+^ transfer rate and shorten the sensor response time (2 s) (Figure 2(b)). The hybrid nanonetwork structure effectively improved the electrochemical stability and sensitivity and solved the noise and signal drift problems in low concentration detection. The sensor was integrated and assembled with the NFC wireless patch system to reduce the device size and energy consumption, while using microchannels to collect sweat from the skin surface to avoid contamination. The device sensitivity can be increased from 63 mV/dec to 173 mV/dec by an integrated amplification system.

Typical sweat collection and sensing technologies are inefficient in extreme situations; for example, there are numerous difficulties in achieving these tasks in water environment. Figure 2(c) illustrates a waterproof epidermal microfluidic electronic sensor that adheres to the skin and collects, stores, and analyzes sweat and operates even in underwater environments [80]. The sensor combines microfluidic technology with electronic functionality, which provides excellent mechanical and barrier properties through the application of flexible styrene block copolymers for the encapsulation of microfluidic systems and devices. The polymer possesses extremely low permeability to water, water vapor, and chemicals in water, thereby eliminating the risk of contamination and preventing the surrounding water from interfering with the sensor. The authors designed the microchannel into a snake shape to accurately measure the volume of sweat that could be accommodated by hydrodynamics to 60 *μ*L. The sensor was able to bond to the skin securely and in a waterproof manner, while maintaining stable monitoring performance, even during extreme underwater operations for more than 2 h.

Multiplexed sensing assists in determining whether a change in the sensing signal originates from the change in the concentration of the target or from other factors. If only the Na^+^ level is monitored independently, it is not possible to determine the factor behind the increased Na^+^ concentration, whether it is the increased rate of sweat secretion or the enhanced Na^+^ level in the body. However, when the sweat secretion rate is also monitored, the specific reason for an increase in Na^+^ content can be determined. For this reason, Baker et al. designed a wearable microfluidic device that can be combined with a smartphone image processing platform to measure localized sweat rate and sweat Cl^−^ concentrations, as shown in Figure 2(d) [81]. The dehydrated orange dye deposited in microchannel 1 enabled rapid visualization of sweat volume and flow rate. Silver chlorobenzoate was used in microchannel 2 as a biodetection reagent for the measurement of Cl^−^ concentration by reacting with Cl^−^ in sweat, yielding purple silver chlorobenzoate. The sensor employed roll-to-roll (R2R) processing of polymeric materials to fabricate a network of microchannels and assay pores, while silver chloranilate solution was added to the assay pores to rapidly measure the sweat rate and Cl^−^ concentration. The sensor investigated the correlation between the regional sweat rate and Cl^−^ concentrations in athletes in a constant environment, as well as during exercise under different environmental conditions. In addition, a smartphone application was able to predict the sweat production rate of the whole body and sweat Cl^−^ concentrations from the regional sweat production rate and sweat Cl^−^ concentrations.

### 4.2. Hospital Monitoring

#### 4.2.1. Drug Monitoring

Medication monitoring is an important part of the doping control and medical process, helping physicians track patient compliance with prescriptions and control the dosage of medications for better treatment. Sweat is an attractive alternative to traditional invasive blood tests, and therefore, wearable sweat biosensors have greatly potential application in future medical treatments.

**Figure 3:**
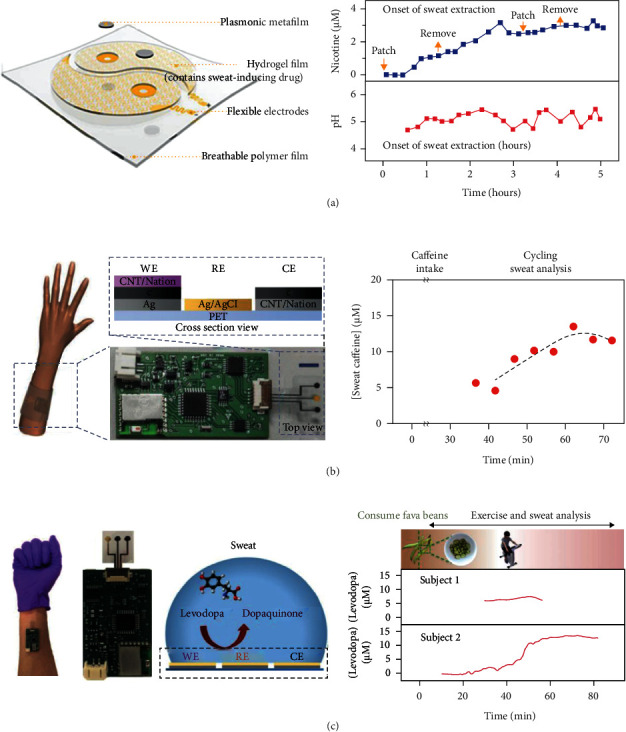
Drug monitoring. (a) Schematic diagram of the sensor array and tracking the pH value and nicotine concentration detection of the forearm. Reproduced with permission from Reference [22], copyright 2021, American Association for the Advancement of Science. (b) Schematic diagram of wearing the flexible sensor patch, S-band optical image, and cross-sectional view and the concentration monitoring of caffeine after ingestion. Reproduced with permission from Reference [83], copyright 2018, Wiley-VCH Verlag GmbH & Co. KGaA, Weinheim. (c) S-band optical image, working mechanism, and electrode cross-sectional view of the worn device and detection of L-dopa sweat concentration after ingesting beans. Reproduced with permission from Reference [84], copyright 2019, American Chemical Society.

The vast majority of electrochemical biosensors lack universal recognition capability because they require the immobilization of specific enzymes or antibodies for specific recognition of the target. Wang et al. employed a surface-enhanced Raman scattering (SERS) sensing element by using an ordered silver nanocube (NC) superlattice. The SERS effect generated by strong electromagnetic fields positioned in the gaps and corners of the NC could be used to detect molecules near the surface of the superfilm (Figure 3(a)) [22]. In addition, the hydrogel was loaded with acetylcholine chloride, which stimulated sweat secretion, and attached to two spiral mesh electrodes using iontophoresis as the sweat extraction element. Importantly, the authors introduced small protective rings to support and protect the NC superlattice in order to maintain the integrity of the ultrafine nanostructure of the SERS sensor during exercise. The proposed wearable sweat biosensor was successfully applied to monitor concentration changes in anesthetics (lidocaine), chemotherapeutic drugs (methotrexate), addictive illicit drugs (cocaine and nicotine), and pH in metabolism. As each molecule possesses a unique SERS “fingerprint” spectrum, the SERS-based wearable sweat biosensor offered high sensitivity and broad specificity for single-molecule detection. The biggest shortcoming of this biosensor is the readout system, which requires a large Raman spectrometer and cannot be monitored in real time or conveniently, but these limitations will be overcome with further development of planar optics.

The drug molecules that are present in low concentration in biological fluids require highly sensitive and specific detection. The most commonly used electrochemical method for drug detection is DPV, which is based on the oxidation of the target molecules at a defined oxidation potential [82]. Tai et al. designed a wearable sweat band for noninvasive and *in situ* monitoring of methylxanthine drugs, and caffeine was chosen as the target for monitoring (Figure 3(b)) [83]. The biosensor consisted of a PET substrate and a three-electrode system connected to a printed circuit board to detect caffeine. The surface of the carbon WE was modified with carbon nanotubes and a Nafion film. Carbon materials were preferred for drug detection because of their stability at high voltages, low cost, and high biocompatibility, while nanotubes provided excellent electrical conductivity and electrocatalysis. The sensitivity of the biosensor to a standard caffeine solution was 110 nA/*μ*M, and the detection limit of the constructed sensor was 3 × 10^−6^ M when the subject ingested espresso and exercised vigorously.

Figure 3(c) illustrated a patch-type wearable sweat biosensor capable of detecting levodopa in sweat [84]. The monitoring and optimization of the dose of levodopa, which is a standard drug for Parkinson's disease patients, are of great importance in treating patients. The sensor was prepared as a three-electrode system on a polyethylene glycol paraben flexible substrate, and the WE surface was modified with gold dendritic nanostructures, which improved the sensitivity and specificity of detection. Subjects consumed 450 g of fava beans containing levodopa in sweat by ionophoresis and exercise sweating, respectively, and researchers found that a peak of 6.6 *μ*M was reached at 47 min and dropped to 3.3 *μ*M at about 74 min, demonstrating the feasibility of the wearable sensor to track levodopa metabolism in real time.

#### 4.2.2. Disease Monitoring

**Figure 4:**
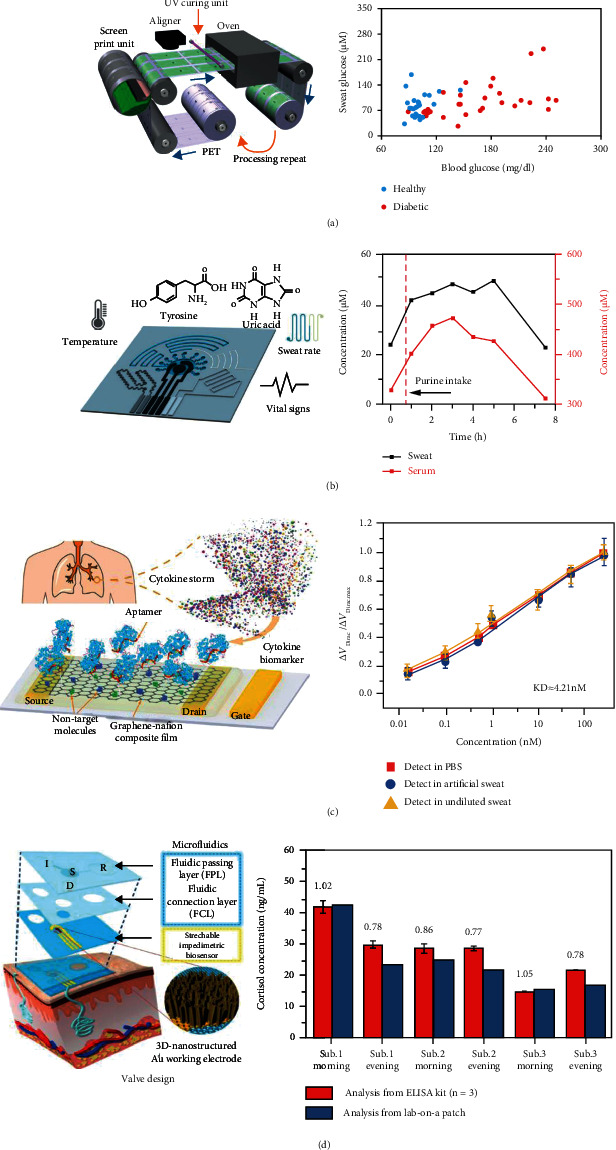
Disease monitoring. (a) Schematic diagram of roll-to-roll rotary screen printing of the wearable biosensing sweat patch and the relationship between the average fasting levels of sweat glucose and blood glucose of healthy subjects and diabetic subjects. Reproduced with permission from Ref. [85], copyright 2019, American Association for the Advancement of Science. (b) Schematic diagram of multiple functions of the sensor: sweat uric acid and tyrosine detection, sweat rate detection, temperature sensing and vital signs monitoring, and dynamic changes of sweat and serum uric acid in subjects before and after the diet (rich in purines). Reproduced with permission from Ref. [86], copyright 2020, Springer Nature. (c) Schematic of the aptameric biosensor for cytokine biomarker detection and the normalized function of different IFN-*γ* concentrations in PBS, artificial sweat, and undiluted sweat. Reproduced with permission from Ref. [39], copyright 2020, Wiley-VCH GmbH. (d) An exploded view of the lap-on-a-patch platform and wearing a patch to test the subject's sweat cortisol level. Reproduced with permission from Ref. [87], copyright 2020, Elsevier B.V.

Sweat concentrations of various metabolites as well as nutrients have been studied for clinical disease prediction, diagnosis, and monitoring. R2R rotary screen printing can be used to manufacture flexible devices in high throughput and at a reduced cost. Laser ablation technology enables not only fast patterning but also the construction of microfluidic channels on flexible substrates. By combining R2R with laser dicing, Nyein et al. developed a mass-producible wearable electrochemical device to achieve the monitoring of multiple metabolites in sweat (Figure 4(a)) [85]. The patch featured a two-layer structure: the first layer was a patterned electrode system for converting analyte concentration and monitoring sweat rate and the second layer was a microfluidic device with a spiral shape, which could be used not only for sweat collection but also for directing the flow of sweat. The wearable patch allowed simultaneous monitoring of sweat parameters, including Na^+^, K^+^, glucose concentration, and sweat rate, in different regions of the body. Finally, the authors successfully monitored the relationship between sweat glucose and blood glucose concentration of healthy individuals and diabetic patients. They also evaluated the relationship between the rate of sweat glucose secretion and blood glucose secretion *via* the constructed sensor.

Gout is the most common form of inflammatory arthritis and is closely interlinked with uric acid and tyrosine levels. Yang et al. adopted a low-cost and large-scale laser processing process technique, thereby designing and preparing a graphene-based wearable electrochemical, temperature, and stress sensor. The resulting sensor was capable of simultaneously monitoring physiological signals, such as respiration and heartbeat during exercise, and achieved accurate noninvasive monitoring of uric acid and amino acids with low concentrations through temperature calibration compensation (Figure 4(b)) [86]. The graphene-based printed electrodes could electrocatalyze the oxidation of uric acid and tyrosine at a specific potential, much more effectively than commercial gold electrodes. The researchers not only evaluated the changes in uric acid and tyrosine concentrations in sweat but also assayed the correlated concentrations of uric acid in blood and sweat of gout patients and healthy individuals, confirming the high correlation between uric acid concentrations in blood and sweat and providing a new avenue for noninvasive monitoring of gout.

Cytokines can be used as biomarkers to monitor trauma, sepsis, cancer, and rheumatic diseases. The development of cytokines capable of continuous monitoring in sweat is of great significance for the monitoring of patients' daily disease conditions. Wang et al. proposed a flexible renewable biosensor capable of accurately detecting the “inflammatory storm” biomarker in sweat (Figure 4(c)) [39]. The sensor was prepared by photolithography, electron beam deposition, and glass photoresist to prepare a metal electrode, followed by chemical vapor deposition (CVD) to prepare a monolayer of graphene on the electrode, and finally a Nafion was added to form an isolation layer. Nafion in the composite membrane protects the graphene and electrodes from contact with interferents, while anchoring the aptamer probe to identify specific biomarkers. The graphene-Nafion composite film was used as the conductive channel of the field-effect transistor, which greatly suppresses the interference of the sensing signal by the nonspecific adsorption on the graphene surface. The composite membrane provided the sensor regenerability, allowing 80 regenerations without electrical failure and maintaining normal operation under a maximum of 100 turns of cyclic crumpling tests. Biomarker concentration monitoring was achieved by measuring the current changes caused by the aptamer-biomarker interaction. The experimental results showed that the constructed sensor was able to rapidly and accurately detect IFN-*γ* in undiluted artificial sweat and real human sweat in the range of 0.015-250 nM with a detection limit of 740 fM.

Cortisol, as a steroid hormone, is considered a mediator between mental stress and health. Cortisol is closely related to posttrauma stress disorder (PTSD) and depression. It is possible to use a wearable sweat sensor to detect cortisol in sweat noninvasively [30]. Lee et al. reported a laboratory wearable lab-on-a-patch (LOP) device for a noninvasive immune assay of cortisol levels (Figure 4(d)) [87]. The sensor was composed of a fluid passage layer (FPL), a fluid connection layer (FCL), and an impedance biosensor. The FPL and FCL were formed by 3D printing PDMS, which consisted of an inlet chamber (I), sensing chamber (S), disposal chamber (D), and reagent chamber (R). The I chamber passively absorbed sweat and transported it to the S chamber. After 15 min of storage in the S chamber, no sweat was secreted. The sweat could therefore be accurately collected for quantitative testing. When the user pushed the button, the electron mediator reagent was injected from R into S, starting the process of redox medium delivery, electrochemical measurement, and washing, sequentially. Antibodies are used as probe biomolecules to detect cortisol in sweat. EIS was performed on the S chamber after reagent injection by calculating the transfer resistance before and after the redox medium solution and converting the cortisol concentration by using the calibration curve. A biosensor patch was then worn to detect sweating cortisol during exercise in the morning and evening. A change curve of cortisol levels in the morning and evening was observed.

### 4.3. Family Monitoring

Nutrients are essential to human health and the maintenance of organ function. In addition to the proper nutrition required according to dietary guidelines, it is also important to monitor the level of nutrition in the human body. With the development of wearable sensor technology, long-term, noninvasive nutrition tracking though wearable biosensors is expected to prevent and manage nutritional imbalances and maintain the nutritional level of the body.

**Figure 5:**
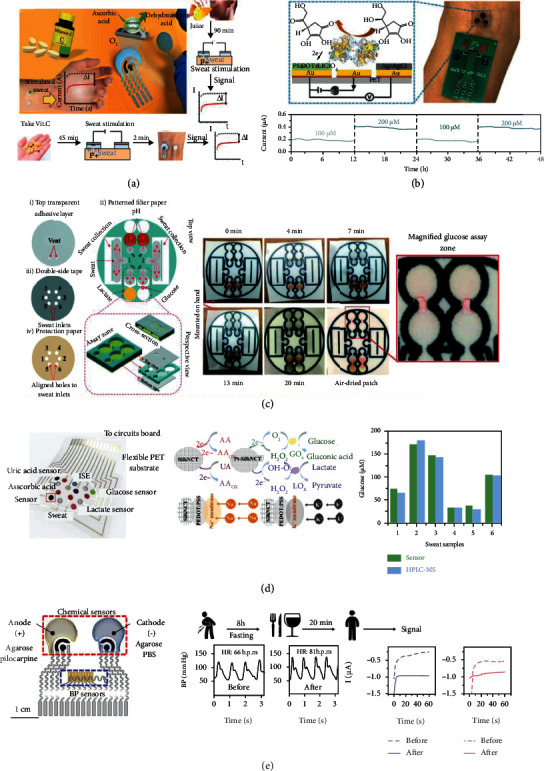
Family monitoring. (a) Schematic diagram of sensor design, working principle, detection process, and current response after taking vitamin C tablets and juice. Reproduced with permission from Ref. [88], copyright 2020, American Chemical Society. (b) Schematic diagram showing the sensing process of the vitamin C sensor and a long-term measurement of a vitamin C sensor. Reproduced with permission from Ref. [89], copyright 2021, Wiley-VCH GmbH. (c) Schematic diagram of device structure design and function and monitoring the glucose and pH and the image of the device after drying. Reproduced with permission from Ref. [46], copyright 2019, The Royal Society of Chemistry. (d) Schematic diagram and working mechanism of multiple electrochemical sensor arrays and comparison of sweat patch sensor and laboratory test of glucose concentration. Reproduced with permission from Ref. [90], copyright 2019, American Association for the Advancement of Science. (e) The acoustic and electrochemical sensing components of the sensor and the blood pressure, alcohol, and glucose sensor signal records before and after food and alcohol intake. Reproduced with permission from Ref. [91], copyright 2021, Springer Nature.

Figure 5(a) illustrates a sweat patch biosensor for the determination of vitamin C in sweat [88]. The sensor was prepared on a polyurethane substrate by combining a WE modified with ascorbate oxidase that could catalyze the oxidation of vitamin C with an ionophoresis system, based on pilocarpine. The concentration of vitamin C was monitored by observing the variations in the redox current, and vitamin C was detected in phosphate buffer solutions from 0-1000 *μ*M. The constructed wearable sweat sensor was also successfully applied to measure the level of vitamin C concentration in human sweat after the intake of juice with vitamin C.

In addition, Zhao et al. selected poly (p-phenylene dimethyl ethylene) ester as the flexible substrate and improved the sensitivity of the sensor by modifying the WE with gold nanodendrites, conducting the polymer poly(3,4-ethylenedioxythiophene) doped with lithium perchlorate (PEDOT : LiClO_4_) and Nafion film layers, which reached a sensitivity of 2.0 nA/*μ*M and a detection limit of approximately 4 *μ*M, exceeding the concentration of vitamin C commonly found in biological fluids (Figure 5(b)) [89]. Subjects wore the sensor for 48 h to obtain prolonged tracking tests, and it was found that the concentration of vitamin C in sweat increased at least 2-fold from a low level (<5 *μ*M) after ingesting 1000 mg of vitamin C within 90 min, indicating that the wearable sweat biosensors can be used for nutrient tracking in sweat to provide guidance for daily diet and personalized health management. The use of enzymes to catalyze the decomposition of the target is a frequently applied method for constructing electrochemical sensors, although enzyme-based electrochemical sensors have problems such as high cost, short storage time, and susceptibility to external factors. Therefore, the development of enzyme free electrochemical sensors is gradually becoming a promising application.

Sweat colorimetric sensing displays offer more convenient visual readings for the target without the need of other complex data processing modules. Zhang et al. prepared a wearable biosensor based on ordinary filter paper and medical tape to measure the pH, glucose, lactate concentration, and overall output of sweat (Figure 5(c)) [46]. Wax printing technology was used to melt printed wax ink through the filter paper and form hydrophobic channels between the hydrophilic cellulose layer and the center. In this way, the filter paper was encapsulated by medical tape. Glucose oxidase, horseradish peroxidase (HRP), 4-aminoantipyrine, and 3,5-dichloro-2-hydroxybenzenesulfonate were used to construct the glucose detection area; lactate oxidase, HRP, and *o*-phenylenediamine dihydrochloride were used to measure lactate; and a universal pH indicator was adopted as the pH detection. The concentration of the target was determined by the intensity of the color after the reaction of the inverse target with a color-developing reagent, and the pH value could be determined by comparison with the standard colorimetric card.

He et al. prepared a silk fabric-derived intrinsically nitrogen- (N-) doped carbon textile- (SilkNCT-) based wristband sweat sensor by using a laser processing strategy for simultaneous monitoring of six biomarkers, including glucose, lactate, ascorbic acid, uric acid, Na^+^, and K^+^ (Figure 5(d)) [90]. The layered and porous structure of S5ilkNCT and the graphitic nanocarbon structure allow SilkNCT to have good electrical conductivity along with good flexibility and better accessibility, improving the electrocatalytic ability of the sensor. Selective detection of glucose and lactate was achieved by uniformly distributing Pt nanospheres on SilkNCT and loading glucose oxidase and lactate oxidase. The detection range of glucose concentration was 25-300 *μ*M with a sensitivity of 6.3 nA/*μ*M and detection limit of 5 *μ*M. The linear range of the lactate sensor was 5-35 mM with a detection limit of 0.5 mM, and the sensitivity was 174.0 nA/mM. Directly using SilkNCT as the working electrode to detect ascorbic acid and uric acid by the DPV method, the results showed that the ascorbic acid biosensor had a linear range of 20-300 *μ*M with a detection limit of 1 *μ*M, and the sensitivity was 22.7 nA/*μ*M. Similarly, the uric acid sensor exhibited a linear response of 2.5-115 *μ*M with a sensitivity of 196.6 nA/*μ*M, and the detection limit was 0.1 *μ*M, and the sensitivity was 196.6 nA/*μ*M. SilkNCT was combined with PEDOT : PSS and ion-selective membranes to selective detect the Na^+^ and K^+^, with detection limits of 1 and 0.5 mM for the Na^+^ and K^+^ sensors, respectively. Six mitomycin-based electrochemical sensors demonstrated their potential to monitor biomarkers, and the integration of signal acquisition and transmission circuit modules allowed the assay results to be received on a smartphone.

The use of noninvasive wearable sensors to monitor individual's physical parameters (e.g., blood pressure and BP) and biochemical parameters (e.g., glucose) has been reported. The integration of physical and chemical sensors in a single device enables the simultaneous monitoring of multiple physiological and biochemical indicators in the human body, facilitating the exploration of correlations between cardiovascular changes and biomarker levels. Based on this, Sempionatto et al. reported a stretchable and integrated wearable sweat biosensor that could simultaneously detect BP, heart rate (HR), and the concentration of glucose, lactate, caffeine, and alcohol (Figure 5(e)) [91]. Researchers have used ultrasound transducers to monitor BP and HR and electrochemical sensors to further measure biomarker levels. The authors successfully prepared wearable integrated sensors with high mechanical flexibility and no sensor crosstalk by integrating rigid and flexible sensor components (custom piezoelectric lead zirconate titanate ultrasound transducers and printed polymer composites) on a stretchable substrate of SEBS through an innovative solvent welding process. The wearable sensor enabled real-time monitoring of ISF, sweat, and cardiovascular parameters. The researchers administered different stimuli (exercise and ingestion of alcohol, food, and caffeine) to the subjects and monitored and assessed the relationship between metabolic changes and hemodynamic activity in response to these stimuli.

## 5. Conclusions and Prospects

Human sweat contains a wealth of biochemical information related to the physical condition, nutritional status, and some diseases of human. The detection of sweat promises to reduce the need for invasive blood tests and enable continuous monitoring of relevant metabolites and related diseases at the molecular level. Therefore, wearable technology for sweat analysis has become an international frontier in the field of wearable devices and has offered remarkable potential for athletes' training adjustments, personal nutritional conditioning, and personalized medical diagnosis. In this review, we focused on the design, preparation, and application of wearable sweat biosensors in different scenarios, in addition to the secretion mechanism of sweat metabolites and some representative components. With the flourishing development of flexible electronics and its integration with different disciplines, noninvasive, continuous, and dynamic monitoring of human health based on sweat metabolites has become available. However, there are still several issues and challenges that need to be addressed before wearable sweat biosensors can truly become a personalized physiological monitoring platform.

Structural design. (1) Improve the sensitivity, selectivity, and stability of the chemical sensors. Most of the materials used in wearable sweat/biosensors for specific detection of metabolites are enzymes, antibodies, and ion-selective membranes. Although these biomaterials can specifically identify the targets, they suffer from limitations such as easy inactivation, poor preservation, and ineffective immobilization. Therefore, the advantages of nanomaterials can be fully utilized to modify the WE surface in a stable and efficient manner. Nanomaterials are available to load more biomaterials (e.g., antibodies, enzymes, and aptamers) to jointly improve the sensitivity of detection. Scientists have developed a number of enzyme-free sensors, but their selectivity needs to be further improved before they are applied to wearable sensing [92–94]. (2) Reduce the size of the power module or adopt a new power supply strategy. At present, commercial button cells are being employed, but they have limitations including large size, heavy weight, and poor mechanical properties. Therefore, flexible batteries, biofuel cells, and frictional self-powered and various other novel power supply methods should be investigated [95–99]. (3) Combine with microfluidics for precise sweat absorption, separation, and simultaneous *in situ* detection of multiple markers and with big data, deep learning, and other technologies for precise, intelligent, and personalized medical prediagnosis. (4) Each structure of wearable sweat biosensors or the miniaturization and integration of the system is still an important barrier. One of the most important issues here is that different sensors are required to be encapsulated to ensure that they have good mechanical capability and anti-interference capability; therefore, further research is needed to implement complex encapsulation strategies to ensure the individual structures can work stably for a long time. Multimodal sensors will provide a more comprehensive picture for personalized medicine, such as integrating physiological signal detection (heartbeat and blood pressure) and biochemical signal detection (glucose and ions) together in one sensor, offering a more comprehensive and accurate understanding of the body's health level. (5) More new designs of electrode and interface structures are needed to be developed to resist motion forgery during the movement of human and obtain more accurate signals.

Material preparation and design. (1) Develop flexible materials or encapsulation materials that are self-healing, highly adhesive, breathable, and resistant to contamination. In practical applications, the human skin may be coated with products such as sunscreen and pollutants in sweat during exercise, which could have an impact on the detection performance. At the same time, the need for long-term wear in chronic disease patients or sports personnel also requires consideration of the breathability and self-healing ability of flexible materials to reduce macroscopic cracks and fractures that will affect the normal function and service life of the material [100–102]. (2) Develop materials that can be applied to extreme environments (e.g., high temperature, high radiation, and high humidity) to serve a variety of application scenarios. For example, in extremely cold or hot environments, the activity of biometric materials (e.g., enzymes and antibodies) is greatly reduced. Therefore, researchers have designed enzyme-free sensors, such as nanomaterials with excellent catalytic and conductive properties that are chemically modified on the surface of a working electrode enabling it to catalyze redox reactions of small molecules (e.g., dopamine and urea) in sweat at a specific bias voltage to generate a current signal. Molecularly imprinted polymers (MIPs) have also been introduced into the design of wearable sweat biosensors. MIPs are a new class of polymeric materials with specific recognition functions, which have binding sites that match the target in terms of functional groups and spatial structure, and importantly, they have the merits of simple preparation and favorable stability and can be used in harsh environments such as acids, bases, high temperatures, and high pressures. Therefore, MIP-based biosensors are expected to solve the problem of antibody biosensor activity in extreme environments. In addition, humans will often face continuously changing environmental temperatures during their activities, such as from hot to cold and from normal room temperature to extreme heat and cold, which also poses a challenge to the durability of materials for wearable biosensors. Researchers have also made numerous efforts in this area; for example, in the design and synthesis of substrate materials, researchers have introduced organic solvents, salts, and ionic liquids into hydrogel substrates to improve the freeze resistance of the materials. (3) The material possesses favorable biocompatibility. Wearable biosensors need to be tightly in contact with the human body for a long time; thus, both the substrate material and the material of the sensing element need to have better biocompatibility in addition to their own inherent properties, which will not cause human skin allergy and other problems in long-term applications [103–105]. The most used flexible substrate materials are based on polymer materials, such as PDMS, but researchers usually introduce some other materials to improve the stretchability and conductivity of flexible substrates, and the biocompatibility of these materials requires significant attention. Some frequently used biocompatibility tests should be applied to the experimental design of wearable biosensors, such as the MTT test, sensitization test, and skin irritation test. In addition, some natural materials can also be used in wearable devices through appropriate modifications, such as silk. These natural materials usually have good biocompatibility, but ductility, Young's modulus, electrical conductivity, etc. are insufficient; therefore, wearable biosensors based on natural materials are also the focus of future development.

With the joint development of flexible electronics and other cross-disciplines, it is anticipated to promote the gradual maturation and commercialization of wearable sweat biosensors in sports, wellness recovery, personalized medical diagnosis, and other fields. Although there are still numerous difficulties to be addressed in the widespread commercial applications of wearable sweat biosensors, we believe that these devices offer great potential in the field of preventative and at-home health monitoring.

## Figures and Tables

**Table 1:** Summary of the detection of biochemical substances in sweat presented in this review.

Analytes	Detection methods	Recognition elements	Preparation technologies	Related diseases	LOD	Application scenarios	References
Na^+^	OCPT	Na^+^ ionophore	Screen printing; laser engraving	Hypernatremia (+); hyponatremia (-)	/; 1 mM	Outdoor monitoring	[16, 90]
Cl^−^	Colorimetry	Silver chloranilate; silver chlorobenzoate	CO_2_ laser manufacturing; roll-to-roll printing	Cystic fibrosis	/	Cystic fibrosis; outdoor monitoring	[80, 81]
K^+^	OCPT	K^+^ ionophore	Screen printing; laser engraving	Hyperkalemia (+); hypokalemia (-)	/; /; 0.5 mM	Outdoor monitoring; family monitoring	[16, 79, 90]
Glucose	CA	Glucose oxidase	Roll-to-roll printing; laser engraving; screen printing	Diabetes (+); hypoglycemia (-)	/; 5 *μ*M; /	Disease monitoring	[85, 90, 91]
Lactate	Colorimetry; CA	Lactate oxidase	Inkjet printing; laser engraving; screen printing	Hypoglycemia; hyperlactatemia (+)	5 mM; 0.5 mM; /	Family monitoring	[46, 90, 91]
Ethanol	CA	Ethanol oxidase	Screen printing	Cirrhosis	/	Family monitoring	[91]
Uric acid	DPV	SilkNCT	Laser engraving	Gout	0.1 *μ*M	Family monitoring	[90]
Ascorbic acid	DPV; SWV; CV	Ascorbic acid oxidase	Laser engraving; screen printing; photolithography and evaporation	Uric acid stones (+); scurvy (-)	/; 4 *μ*M; 1 *μ*M	Family monitoring	[88, 89, 90]
L-Dopamine	DPV	Gold dendritic nanostructures	Photolithography and electron beam evaporation	Parkinson's disease	1.25 *μ*M	Drug monitoring	[84]
Tyrosine	DPV	Gold electrode	Laser engraving	Tyrosinemia	3.6 *μ*M	Health monitoring	[86]
Caffeine	DPV	Multiwalled carbon nanotubes	Roll-to-roll printing; screen printing	Coronary syndrome	3 *μ*M; /	Drug monitoring; family monitoring	[83, 91]
Cortisol	CV	Cortisol antibody	3D printing	Depressive disorder	1 pg/mL	Disease monitoring	[87]
IFN-*γ*	Time-resolved measurement	Aptamer	Photolithography and electron beam evaporation	Autoimmune disease	740 fM	Disease monitoring	[39]
Nicotine	SERS spectrum	Silver nanocube superlattice	Laser direct writing technique	Nicotine poisoning	0.01 nM	Drug monitoring	[22]

(+): increased concentration; (-): reduced concentration.

## References

[1] Bariya M., Nyein H. Y. Y., Javey A. (2018). Wearable sweat sensors. *Nature Electronics*.

[2] Bandodkar A. J., Jeang W. J., Ghaffari R., Rogers J. A. (2019). Wearable sensors for biochemical sweat analysis. *Annual Review of Analytical Chemistry*.

[3] Li Z., Zheng Q., Wang Z. L., Li Z. (2020). Nanogenerator-based self-powered sensors for wearable and implantable electronics. *Research*.

[4] Kim J., Chou E. F., le J., Wong S., Chu M., Khine M. (2019). Soft wearable pressure sensors for beat-to-beat blood pressure monitoring. *Advanced Healthcare Materials*.

[5] Xu L., Zhang Z., Gao F. (2021). Self-powered ultrasensitive pulse sensors for noninvasive multi-indicators cardiovascular monitoring. *Nano Energy*.

[6] Gao W., Emaminejad S., Nyein H. Y. Y. (2016). Fully integrated wearable sensor arrays for multiplexed _in situ_ perspiration analysis. *Nature*.

[7] Koh A., Kang D., Xue Y. (2016). A soft, wearable microfluidic device for the capture, storage, and colorimetric sensing of sweat. *Science Translational Medicine*.

[8] Elsherif M., Hassan M. U., Yetisen A. K., Butt H. (2018). Wearable contact lens biosensors for continuous glucose monitoring using smartphones. *ACS Nano*.

[9] Chen Y., Lu S., Zhang S. (2017). Skin-like biosensor system via electrochemical channels for noninvasive blood glucose monitoring. *Science Advances*.

[10] Heikenfeld J., Jajack A., Feldman B. (2019). Accessing analytes in biofluids for peripheral biochemical monitoring. *Nature Biotechnology*.

[11] Koydemir H. C., Ozcan A. (2018). Wearable and implantable sensors for biomedical applications. *Annual Review of Analytical Chemistry*.

[12] Ghaffari R., Rogers J. A., Ray T. R. (2021). Recent progress, challenges, and opportunities for wearable biochemical sensors for sweat analysis. *Sensors and Actuators B: Chemical*.

[13] Brothers M. C., DeBrosse M., Grigsby C. C. (2019). Achievements and challenges for real-time sensing of analytes in sweat within wearable platforms. *Accounts of Chemical Research*.

[14] Parrilla M., Guinovart T., Ferré J., Blondeau P., Andrade F. J. (2019). A wearable paper-based sweat sensor for human perspiration monitoring. *Advanced Healthcare Materials*.

[15] Xu H., Lu Y. F., Xiang J. X. (2018). A multifunctional wearable sensor based on a graphene/inverse opal cellulose film for simultaneous in situ monitoring of human motion and sweat. *Nanoscale*.

[16] Alizadeh A., Burns A., Lenigk R. (2018). A wearable patch for continuous monitoring of sweat electrolytes during exertion. *Lab on a Chip*.

[17] Yang Y., Gao W. (2019). Wearable and flexible electronics for continuous molecular monitoring. *Chemical Society Reviews*.

[18] Hu Y., Converse C., Lyons M. C., Hsu W. H. (2018). Neural control of sweat secretion: a review. *British Journal of Dermatology*.

[19] Ge Y., Wei P., Wang T., Cao X., Zhang D., Li F. (2018). A simple fluorescent probe for monitoring pH in cellsbased on new fluorophorepyrido[1,2 _-a_ ]benzimidazole. *Sensors and Actuators B: Chemical*.

[20] Guo Y., Werner C. F., Handa S. (2021). Miniature multiplexed label-free pH probe _in vivo_. *Biosensors and Bioelectronics*.

[21] Wang L., Wang L., Zhang Y. (2018). Weaving sensing fibers into electrochemical fabric for real-time health monitoring. *Advanced Functional Materials*.

[22] Wang Y., Zhao C., Wang J. (2021). Wearable plasmonic-metasurface sensor for noninvasive and universal molecular fingerprint detection on biointerfaces. *Science Advances*.

[23] An Y. H., Lee J., Son D. U. (2020). Facilitated transdermal drug delivery using nanocarriers-embedded electroconductive hydrogel coupled with reverse electrodialysis-driven iontophoresis. *ACS Nano*.

[24] Simmers P., Li S. K., Kasting G., Heikenfeld J. (2018). Prolonged and localized sweat stimulation by iontophoretic delivery of the slowly-metabolized cholinergic agent carbachol. *Journal of Dermatological Science*.

[25] Mena-Bravo A., Luque de Castro M. D. (2014). Sweat: a sample with limited present applications and promising future in metabolomics. *Journal of Pharmaceutical and Biomedical Analysis*.

[26] Sonner Z., Wilder E., Heikenfeld J. (2015). The microfluidics of the eccrine sweat gland, including biomarker partitioning, transport, and biosensing implications. *Biomicrofluidics*.

[27] Abellán-Llobregat A., Jeerapan I., Bandodkar A. (2017). A stretchable and screen-printed electrochemical sensor for glucose determination in human perspiration. *Biosensors and Bioelectronics*.

[28] Gao B., Elbaz A., He Z. (2018). Bioinspired kirigami fish-based highly stretched wearable biosensor for human biochemical-physiological hybrid monitoring. *Advanced Materials Technologies*.

[29] Ardalan S., Hosseinifard M., Vosough M., Golmohammadi H. (2020). Towards smart personalized perspiration analysis: An IoT-integrated cellulose- based microfluidic wearable patch for smartphone fluorimetric multi-sensing of sweat biomarkers. *Biosensors and Bioelectronics*.

[30] Cheng C., Li X., Xu G. (2021). Battery-free, wireless, and flexible electrochemical patch for _in situ_ analysis of sweat cortisol via near field communication. *Biosensors and Bioelectronics*.

[31] Kim S. B., Koo J., Yoon J. (2020). Soft, skin-interfaced microfluidic systems with integrated enzymatic assays for measuring the concentration of ammonia and ethanol in sweat. *Lab on a Chip*.

[32] Pirovano P., Dorrian M., Shinde A. (2020). A wearable sensor for the detection of sodium and potassium in human sweat during exercise. *Talanta*.

[33] Anastasova S., Crewther B., Bembnowicz P. (2017). A wearable multisensing patch for continuous sweat monitoring. *Biosensors and Bioelectronics*.

[34] Kim J., Lee S., Kim S., Jung M., Lee H., Han M. S. (2020). Development of a fluorescent chemosensor for chloride ion detection in sweat using Ag^+^-benzimidazole complexes. *Dyes and Pigments*.

[35] Gao W., Nyein H. Y. Y., Shahpar Z. (2016). Wearable microsensor array for multiplexed heavy metal monitoring of body fluids. *ACS Sensors*.

[36] Wu M. J., Hu H. H., Siao C. Z. (2018). All organic label-like copper(II) ions fluorescent film sensors with high sensitivity and stretchability. *ACS Sensors*.

[37] Macleod T., Ward J., Alase A. A., Bridgewood C., Wittmann M., Stonehouse N. J. (2019). Antimicrobial peptide LL-37 facilitates intracellular uptake of RNA aptamer Apt 21-2 without inducing an inflammatory or interferon response,. *Frontiers in Immunology*.

[38] Zhao X., Gao Y., Wang J. (2020). Aggregation-induced emission based one-step “lighting up” sensor array for rapid protein identification. *Chemical Communications*.

[39] Wang Z., Hao Z., Wang X. (2021). A flexible and regenerative aptameric graphene-nafion biosensor for cytokine storm biomarker monitoring in undiluted biofluids toward wearable applications. *Advanced Functional Materials*.

[40] Zhang C., Kim J. P., Creer M., Yang J., Liu Z. W. (2017). A smartphone-based chloridometer for point-of-care diagnostics of cystic fibrosis. *Biosensors and Bioelectronics*.

[41] Ghaffari R., Choi J., Raj M. S. (2020). Soft wearable systems for colorimetric and electrochemical analysis of biofluids. *Advanced Functional Materials*.

[42] Chen A., Chatterjee S. (2013). Nanomaterials based electrochemical sensors for biomedical applications. *Chemical Society Reviews*.

[43] Yu Y., Nyein H. Y. Y., Gao W., Javey A. (2020). Flexible electrochemical bioelectronics: the rise of in situ bioanalysis. *Advanced Materials*.

[44] Ding J., Qin W. (2020). Recent advances in potentiometric biosensors. *TrAC Trends in Analytical Chemistry*.

[45] Luo X., Davis J. J. (2013). Electrical biosensors and the label free detection of protein disease biomarkers. *Chemical Society Reviews*.

[46] Zhang Z., Azizi M., Lee M., Davidowsky P., Lawrence P., Abbaspourrad A. (2019). A versatile, cost-effective, and flexible wearable biosensor for in situ and ex situ sweat analysis, and personalized nutrition assessment. *Lab on a Chip*.

[47] Sekine Y., Kim S. B., Zhang Y. (2018). A fluorometric skin-interfaced microfluidic device and smartphone imaging module for in situ quantitative analysis of sweat chemistry. *Lab on a Chip*.

[48] Choi J., Bandodkar A. J., Reeder J. T. (2019). Soft, skin-integrated multifunctional microfluidic systems for accurate colorimetric analysis of sweat biomarkers and temperature. *ACS Sensors*.

[49] Xu X. Y., Yan B. (2018). A fluorescent wearable platform for sweat Cl−analysis and logic smart-device fabrication based on color adjustable lanthanide MOFs. *Journal of Materials Chemistry C*.

[50] Kim S. B., Zhang Y., Won S. M. (2018). Super-absorbent polymer valves and colorimetric chemistries for time-sequenced discrete sampling and chloride analysis of sweat via skin-mounted soft microfluidics. *Small*.

[51] Bandodkar A. J., Gutruf P., Choi J. (2019). Battery-free, skin-interfaced microfluidic/electronic systems for simultaneous electrochemical, colorimetric, and volumetric analysis of sweat. *Science Advances*.

[52] Gao Y., Yu L., Yeo J. C., Lim C. T. (2020). Flexible hybrid sensors for health monitoring: materials and mechanisms to render wearability. *Advanced Materials*.

[53] Song J., Feng X., Huang Y. (2016). Mechanics and thermal management of stretchable inorganic electronics. *National Science Review*.

[54] Wang X., Liu Z., Zhang T. (2017). Flexible sensing electronics for wearable/attachable health monitoring. *Small*.

[55] Yeo W. H., Kim Y. S., Lee J. (2013). Multifunctional epidermal electronics printed directly onto the skin. *Advanced Materials*.

[56] Li S., Wang J., Peng W. (2017). Sustainable energy source for wearable electronics based on multilayer elastomeric triboelectric nanogenerators. *Advanced Energy Materials*.

[57] Yan Z., Han M., Shi Y. (2017). Three-dimensional mesostructures as high-temperature growth templates, electronic cellular scaffolds, and self-propelled microrobots. *Proceedings of the National Academy of Sciences of the United States of America*.

[58] Ma Z., Li S., Wang H. (2019). Advanced electronic skin devices for healthcare applications. *Journal of Materials Chemistry B*.

[59] Tao X., Liao S., Wang Y. (2021). Polymer‐assistedfully recyclable flexible sensors. *EcoMat*.

[60] Hou X., Zhu J., Qian J. (2018). Stretchable triboelectric textile composed of wavy conductive-cloth PET and patterned stretchable electrode for harvesting multivariant human motion energy. *ACS Applied Material Interfaces*.

[61] Eduok U., Faye O., Szpunar J. (2017). Recent developments and applications of protective silicone coatings: a review of PDMS functional materials. *Progress in Organic Coatings*.

[62] Promphet N., Hinestroza J. P., Rattanawaleedirojn P. (2020). Cotton thread-based wearable sensor for non-invasive simultaneous diagnosis of diabetes and kidney failure. *Sensors and Actuators B: Chemical*.

[63] Fu Q., Cui C., Meng L., Hao S., Dai R., Yang J. (2021). Emerging cellulose-derived materials: a promising platform for the design of flexible wearable sensors toward health and environment monitoring. *Materials Chemistry Frontiers*.

[64] Gong M. M., Sinton D. (2017). Turning the page: advancing paper-based microfluidics for broad diagnostic application. *Chemical Reviews*.

[65] Zhang D., Zhang Y., Lu W. (2019). Fluorescent hydrogel-coated paper/textile as flexible chemosensor for visual and wearable mercury (II) detection. *Advanced Materials Technologies*.

[66] Lai C. W., Yu S. S. (2020). 3D printable strain sensors from deep eutectic solvents and cellulose nanocrystals. *ACS Applied Material Interfaces*.

[67] Tu H., Zhu M., Duan B., Zhang L. (2021). Recent progress in high-strength and robust regenerated cellulose materials. *Advanced Materials*.

[68] Cho S. Y., Yu H., Choi J. (2019). Continuous meter-scale synthesis of weavable tunicate cellulose/carbon nanotube fibers for high-performance wearable sensors. *ACS Nano*.

[69] Ray T. R., Choi J., Bandodkar A. J. (2019). Bio-integrated wearable systems: a comprehensive review. *Chemical Reviews*.

[70] Wang Q., Pan X., Guo J. (2021). Lignin and cellulose derivatives-induced hydrogel with asymmetrical adhesion, strength, and electriferous properties for wearable bioelectrodes and self- powered sensors. *Chemical Engineering Journal*.

[71] Lipomi D. J., Lee J. A., Vosgueritchian M., Tee B. C. K., Bolander J. A., Bao Z. (2012). Electronic properties of transparent conductive films of PEDOT:PSS on stretchable substrates. *Chemistry of Materials*.

[72] Lei Z., Wu P. (2018). A supramolecular biomimetic skin combining a wide spectrum of mechanical properties and multiple sensory capabilities. *Nature Communications*.

[73] Park Y. G., Lee S., Park J. U. (2019). Recent progress in wireless sensors for wearable electronics. *Sensors*.

[74] Haartsen J. (2018). How we made Bluetooth. *Nature Electronics*.

[75] Jeong H., Wang L., Ha T. (2019). Modular and reconfigurable wireless e-tattoos for personalized sensing. *Advanced Materials Technologies*.

[76] Kim J., Campbell A. S., de Avila B. E. F., Wang J. (2019). Wearable biosensors for healthcare monitoring. *Nature Biotechnology*.

[77] Liu G., Gao Y., Xu S. (2021). One-stop fabrication of triboelectric nanogenerator based on 3D printing.

[78] Seshadri D. R., Li R. T., Voos J. E. (2019). Wearable sensors for monitoring the physiological and biochemical profile of the athlete. *npj Digital Medicine*.

[79] Zhang S., Zahed M. A., Sharifuzzaman M. (2021). A wearable battery-free wireless and skin-interfaced microfluidics integrated electrochemical sensing patch for on-site biomarkers monitoring in human perspiration. *Biosensors and Bioelectronics*.

[80] Reeder J. T., Choi J., Xue Y. (2019). Waterproof, electronics-enabled, epidermal microfluidic devices for sweat collection, biomarker analysis, and thermography in aquatic settings. *Science Advances*.

[81] Baker L. B., Model J. B., Barnes K. A. (2020). Skin-interfaced microfluidic system with personalized sweating rate and sweat chloride analytics for sports science applications. *Science Advances*.

[82] Muti M., Sharma S., Erdem A., Papakonstantinou P. (2011). Electrochemical monitoring of nucleic acid hybridization by single-use graphene oxide-based sensor. *Electroanalysis*.

[83] Tai L. C., Gao W., Chao M. (2018). Methylxanthine drug monitoring with wearable sweat sensors. *Advanced Materials*.

[84] Tai L. C., Liaw T. S., Lin Y. (2019). Wearable sweat band for noninvasive levodopa monitoring. *Nano Letters*.

[85] Nyein H. Y. Y., Bariya M., Kivimäki L. (2019). Regional and correlative sweat analysis using high-throughput microfluidic sensing patches toward decoding sweat. *Science Advances*.

[86] Yang Y., Song Y., Bo X. (2020). A laser-engraved wearable sensor for sensitive detection of uric acid and tyrosine in sweat. *Nature Biotechnology*.

[87] Lee H. B., Meeseepong M., Trung T. Q., Kim B. Y., Lee N. E. (2020). A wearable lab-on-a-patch platform with stretchable nanostructured biosensor for non-invasive immunodetection of biomarker in sweat. *Biosensors and Bioelectronics*.

[88] Sempionatto J. R., Khorshed A. A., Ahmed A. (2020). Epidermal enzymatic biosensors for sweat vitamin C: toward personalized nutrition. *ACS Sensors*.

[89] Zhao J., Nyein H. Y. Y., Hou L. (2021). A wearable nutrition tracker. *Advanced Materials*.

[90] He W., Wang C., Wang H. (2019). Integrated textile sensor patch for real-time and multiplex sweat analysis. *Science Advances*.

[91] Sempionatto J. R., Lin M., Yin L. (2021). An epidermal patch for the simultaneous monitoring of haemodynamic and metabolic biomarkers. *Nature Biomedical Engineering*.

[92] Adeel M., Rahman M. M., Caligiuri I., Canzonieri V., Rizzolio F., Daniele S. (2020). Recent advances of electrochemical and optical enzyme-free glucose sensors operating at physiological conditions. *Biosensors and Bioelectronics*.

[93] Tang W., Yin L., Sempionatto J. R., Moon J. M., Teymourian H., Wang J. (2021). Touch-based stressless cortisol sensing. *Advanced Materials*.

[94] Chen M. M., Cheng S. B., Ji K. (2019). Construction of a flexible electrochemiluminescence platform for sweat detection. *Chemical Science*.

[95] Sun T., Shen L., Jiang Y. (2020). Wearable textile supercapacitors for self-powered enzyme-free smartsensors. *ACS Applied Material Interfaces*.

[96] Zheng S., Wang H., Das P. (2021). Multitasking MXene inks enable high-performance printable microelectrochemical energy storage devices for all-flexible self-powered integrated systems. *Advanced Materials*.

[97] Yu Y., Nassar J., Xu C. (2020). Biofuel-powered soft electronic skin with multiplexed and wireless sensing for human-machine interfaces. *Science Robotics*.

[98] Wang Z., Li X., Yang Z. (2021). Fully transient stretchable fruit-based battery as safe and environmentally friendly power source for wearable electronics. *EcoMat*.

[99] Yang B., Xiong Y., Ma K., Liu S., Tao X. (2020). Recent advances in wearable textile-based triboelectric generator systems for energy harvesting from human motion. *EcoMat*.

[100] Ge G., Lu Y., Qu X. (2020). Muscle-inspired self-healing hydrogels for strain and temperature sensor. *ACS Nano*.

[101] Pei X., Zhang H., Zhou Y., Zhou L., Fu J. (2020). Stretchable, self-healing and tissue-adhesive zwitterionic hydrogels as strain sensors for wireless monitoring of organ motions. *Materials Horizons*.

[102] Khatib M., Zohar O., Haick H. (2021). Self-healing soft sensors: from material design to implementation. *Advanced Materials*.

[103] Choi S., Han S. I., Jung D. (2018). Highly conductive, stretchable and biocompatible Ag-Au core-sheath nanowire composite for wearable and implantable bioelectronics. *Nature Nanotechnology*.

[104] Adak A., Ghosh S., Gupta V., Ghosh S. (2019). Biocompatible lipopeptide-based antibacterial hydrogel. *Biomacromolecules*.

[105] Wang C., Yokota T., Someya T. (2021). Natural biopolymer-based biocompatible conductors for stretchable bioelectronics. *Chemical Reviews*.

